# Tanshinone IIA Inhibits the Endoplasmic Reticulum Stress-Induced Unfolded Protein Response by Activating the PPARα/FGF21 Axis to Ameliorate Nonalcoholic Steatohepatitis

**DOI:** 10.3390/antiox13091026

**Published:** 2024-08-23

**Authors:** Dajin Pi, Zheng Liang, Jinyue Pan, Jianwei Zhen, Chuiyang Zheng, Wen Fan, Qingliang Song, Maoxing Pan, Qinhe Yang, Yupei Zhang

**Affiliations:** School of Traditional Chinese Medicine, Jinan University, Guangzhou 510632, China; pdj9642@stu2019.jnu.edu.cn (D.P.); liangzheng@stu2021.jnu.edu.cn (Z.L.); pjyflww@stu.jnu.edu.cn (J.P.); zjw1999@stu2022.jnu.edu.cn (J.Z.); chuiyangz@stu2018.jnu.edu.cn (C.Z.); fanww1999@stu2022.jnu.edu.cn (W.F.); sql1207@stu2020.jnu.edu.cn (Q.S.)

**Keywords:** Tanshinone IIA, nonalcoholic steatohepatitis, endoplasmic reticulum, unfolded protein response, PPARα/FGF21 axis

## Abstract

Nonalcoholic steatohepatitis (NASH) is a critical stage in the progression of nonalcoholic fatty liver disease (NAFLD). Tanshinone IIA (TIIA) is a tanshinone extracted from Salvia miltiorrhiza; due to its powerful anti-inflammatory and antioxidant biological activities, it is commonly used for treating cardiovascular and hepatic diseases. A NASH model was established by feeding mice a methionine and choline-deficient (MCD) diet. Liver surface microblood flow scanning, biochemical examination, histopathological examination, cytokine analysis through ELISA, lipidomic analysis, transcriptomic analysis, and Western blot analysis were used to evaluate the therapeutic effect and mechanism of TIIA on NASH. The results showed that TIIA effectively reduced lipid accumulation, fibrosis, and inflammation and alleviated endoplasmic reticulum (ER) stress. Lipidomic analysis revealed that TIIA normalized liver phospholipid metabolism in NASH mice. A KEGG analysis of the transcriptome revealed that TIIA exerted its effect by regulating the PPAR signalling pathway, protein processing in the ER, and the NOD-like receptor signalling pathway. These results suggest that TIIA alleviates NASH by activating the PPARα/FGF21 axis to negatively regulate the ER stress-induced unfolded protein response (UPR).

## 1. Introduction

The course of nonalcoholic fatty liver disease (NAFLD) ranges from isolated steatosis to severe nonalcoholic steatohepatitis (NASH). The former has a relatively benign clinical course and no progression, and NASH is characterized by hepatocyte damage, inflammation, and fibrosis that may even lead to cirrhosis and hepatocellular carcinoma [1]. Studies have shown that the prevalence of NASH ranges from 1.5% to 6.5% and that the prevalence is expected to double by 2030 [2]. The pathogenesis of NASH is unclear and may be related to oxidative stress, the inflammatory response, mitochondrial damage, intestinal flora disorders, and endoplasmic reticulum (ER) stress [3,4,5]. The recommended treatment for NASH remains limited to lifestyle modifications [6,7]. Therefore, identifying natural remedies for the prevention of NASH without side effects has become an urgent endeavour.

Traditional Chinese medicine (TCM) is characterized by multi-target and multi-pathway mechanisms of action, with well-known curative effects. In recent years, a variety of TCM ingredients have been confirmed to have hepatoprotective, anti-inflammatory, and antioxidant activities [8,9]. Due to its potent anti-inflammatory and antioxidant biological activities, Salvia miltiorrhiza is widely used in Asia to treat cardiovascular and liver diseases [10,11,12,13,14]. Tanshinone IIA (TIIA) is a tanshinone extracted from Salvia miltiorrhiza. TIIA has been shown to reduce inflammation and fibrosis in a mouse model of NASH [15,16]. TIIA has also been shown to reduce palmitic acid-induced apoptosis by inhibiting ER stress in HepG2 hepatocytes [17]. TIIA may be a potential drug for the treatment of NASH.

The ER is an important site for lipid synthesis [18]. ER homeostasis is essential for maintaining the normal physiological function of cells [19,20]. Free fatty acids, hyperglycaemia, oxidative stress, calcium imbalance, and other factors can disrupt the ER homeostasis of hepatocytes, leading to ER stress, thereby activating the unfolded protein response (UPR) [21,22,23]. During the occurrence and development of NAFLD, chronic ER stress caused by a high-fat diet continuously stimulates the UPR, promotes hepatic lipid synthesis, inhibits the decomposition and excretion of lipids in hepatocytes, and thus exacerbates hepatic fat accumulation [24]. Therefore, improving ER stress plays a key role in alleviating the progression of NAFLD. A protective effect of TIIA on ER stress in NASH mice has not yet been reported. In this study, a mouse model of NASH was established to systematically investigate the effects of TIIA on NASH.

## 2. Materials and Methods

### 2.1. Materials and Reagents

TIIA (CAS No.568-72-9, purity ≥ 98%) was purchased from Shanghai Aladdin Biochemical Technology Co., Ltd. (Shanghai, China). Polyene phosphatidylcholine capsules (PPCs) were obtained from Sanofi (Beijing, China) Pharmaceutical Co., Ltd.. Assay kits for the measurement of total cholesterol (TC), triglycerides (TG), nonesterified fatty acids (NEFAs), high-density lipoprotein cholesterol (HDL-C), low-density lipoprotein cholesterol (LDL-C), alanine aminotransferase (ALT), aspartate aminotransferase (AST), superoxide dismutase (SOD), malondialdehyde (MDA), and glutathione (GSH) were purchased from Nanjing Jiancheng Bioengineering (NJJCBIO) Institute (Nanjing, China). ELISA kits were purchased from MultiSciences (Lianke) Biotechnology Corporation Limited (Hangzhou, China). Cell Signaling Technology (Massachusetts, MA, USA) provided antibodies for NLRP3, ASC, Caspase-1, and β-Actin. The antibodies against FGF21, BiP, IRE1, PERK, and ATF6 were obtained from Bioss Antibodies (Beijing, China), while anti-PPARα was obtained from Abcam (Cambridge, MA, USA). We obtained HE, Oil Red O, and Masson staining kits from Servicebio Technology Co., Ltd. (Wuhan, China).

Trophic Animal Feed High-tech Co., Ltd. (Nantong, China), provided the L-amino acid diet, which contains 60 kcal % fat, 0.1% methionine, and no additional choline (No. TP 36225MCD). The MCS diet, matches to the MCD diet, includes an L-amino acid diet that has 10 kca % fat and contains methionine and choline. This diet was also provided by Trophic Animal Feed High-tech Co., Ltd. (No. TP 36225MCS).

### 2.2. Establishment of the NASH Model and Treatments

Five-week-old male C57BL/6J mice were acquired from HFK Biochemical Technology Co., Ltd. (Beijing, China) The research was carried out at the Institute of Laboratory Animal Science, Jinan University (Guangzhou, China), following the appropriate experimental protocols (Animal Ethics No. IACUC-20220114-06). The mice were randomly grouped and kept in a controlled environment with regulated temperature and humidity, following a 12 h cycle of light and darkness. Throughout the experiment, all groups had unrestricted availability of nourishment and hydration.

After acclimation for 10 days, fifty mice were randomly divided into five groups according to body weight (*n* = 10 each): MCS group, MCD group, MCD + TIIA-L group, MCD + TIIA-H group, and MCD + PPC group. The mice in the MCD, MCD + TIIA-L, MCD + TIIA-H, and MCD + PPC groups were fed a methionine- and choline-deficient diet for 3 weeks, and the mice in the MCS group were fed a methionine- and choline-sufficient diet for 3 weeks. All mice received oral gavage daily. The MCD + TIIA-L group received TIIA (10 mg/kg/day), the MCD + TIIA-H group received TIIA (20 mg/kg/day), the MCD + PPC group received PPC (120 mg/kg/day), and the MCS and MCD groups received an equivalent volume of deionized water.

### 2.3. Liver Surface Microblood Flow Scan

After 12 h of fasting, mice were anesthetized through an intraperitoneal injection of pentobarbital (50 mg/kg), and the liver was exposed for blood flow measurement. Blood flow volume on the surface of the liver was measured, and a visual blood flow profile was generated using a moorFLPI-2 Full-Field Laser Perfusion Imager Moor Instruments (Axminster, UK) at room temperature and normal light. The angle between the scan head and the liver surface was 90°, and the distance was approximately 20 cm. The data were recorded at 1 frame per second for 60 s.

### 2.4. Biochemical Analysis

The blood samples were subjected to centrifugation at a temperature of 4 °C and a speed of 3600 rpm for 15 min. The serum biomarkers ALT, AST, HDL-C, and LDL-C were then assessed using kits following the procedures of manufacturers. The liver specimens were weighed, homogenized, and subsequently centrifuged to obtain supernatants from the homogenates. Afterward, the levels of TC, TG, and NEFA in the liver homogenates were evaluated by utilizing the corresponding TC, TG, and NEFA detection kits.

### 2.5. Cytokine Analysis Using ELISAs

Part of the liver tissues in each group were homogenized with lysis buffer to extract total protein. The homogenate was centrifuged at 12,000× *g* at 4 °C for 15 min to collect the supernatant. The levels of IL-1β, IL-6, and TNF-α in the tissues were measured using ELISA kits according to the manufacturer’s instructions and quantified according to the standard curve. All experiments were repeated six times and the levels of cytokines were expressed in pg/mL.

### 2.6. Detection of Oxidative Stress Indexes

Homogenization was performed using the sample homogenization buffer provided with the kit. Subsequently, the homogenate was centrifuged at 12,000 rpm for 10 min. The supernatant was collected, and the levels of SOD, MDA, and GSH in the supernatant were measured according to the instructions of the assay kit. All experiments were repeated six times.

### 2.7. Histopathological Examination

Samples of liver tissue were obtained after death and promptly preserved in 4% paraformaldehyde for histopathological analysis. The preserved tissues were encased in paraffin, sliced into 5 micron thick sections, and subjected to HE staining for overall histological assessment. To assess lipid accumulation, tissue sections underwent staining using Oil Red O, a specific dye for neutral lipids. Masson and α-SMA were used to evaluate fibrosis. To assess macrophage infiltration, immunofluorescence (IF) staining was performed using antibodies against F4/80, CD68 (cluster of differentiation 68) and CD206 (cluster of differentiation 206). Fluorescently labelled samples were examined using a microscope to observe areas with discoloration. In addition, we performed DHE staining in strict adherence to the manufacturer’s instructions to assess the levels of reactive oxygen species (ROS) in liver tissues. ROS levels were measured by capturing the fluorescence intensity of DHE using a fluorescence microscope and quantified with image analysis software. To conduct thorough examinations of liver tissue at the microscopic level, we immediately stored small liver sections in a solution containing 2.5% glutaraldehyde buffered with 0.1 M phosphate (pH 7.4). Following rinses in phosphate buffer, the specimens underwent postfixation in 1% osmium tetroxide. Subsequent steps included dehydration through an incremental ethanol series and embedding within EPON resin. Thin slices, approximately 70 nm thick, were created using an ultramicrotome, placed on copper mesh grids, and then stained with uranyl acetate and lead citrate. We utilized Transmission Electron Microscopy (TEM) (TECNAI G2 SPIRIT TWINE, FEI) to observe the changes in the cellular structure of liver cells, paying special attention to the overall condition of the ER.

### 2.8. Liver Lipidomic Analysis

A glass tube with a Teflon-lined cap was used to vortex 0.75 mL of methanol with 100 mg of liver tissue powder. Afterward, we added 2.5 mL of MTBE to the mixture and allowed it to incubate for 1 h at room temperature while stirring. To achieve phase separation, 0.625 mL of MS-grade water was incorporated. Following a 10 min incubation at room temperature, centrifugation was carried out at 1000× *g* for a duration of 10 min. The upper organic layer was carefully retrieved, while the residual aqueous phase underwent a secondary extraction using 1 mL of a solvent combination (MTBE/methanol/water at a 10:3:2.5 volume ratio). The organic layer from this secondary extraction was combined with the first fraction. The combined natural mixture was subsequently dried and reconstituted in 100 μL of isopropanol for conservation. The lipid composition was examined by utilizing a UHPLC system connected to a high-resolution MS/MS instrument for lipidomic analysis. To cover a wide range of lipid categories, we utilized both positive and negative ion modes in the mass spectrometer. Proprietary software was utilized to process the raw data, enabling peak detection and alignment. Lipid identification was performed by comparing their precise mass, retention time, and MS/MS spectra with a reference lipid database. Lipid quantification was conducted by normalizing peak areas. By employing various multivariate statistical techniques, such as principal component analysis (PCA) and partial least squares-discriminant analysis (PLS-DA), we were able to detect lipids with differing levels of abundance across different groups. Lipid metabolites that were considered differentially altered (DALs) met the following criteria: a variable importance in projection (VIP) greater than 1, a *p* value less than 0.05, and a fold change (FC) either equal to or greater than 2 or equal to or less than 0.5.

### 2.9. Liver Transcriptomic Analysis

For subsequent analysis, only samples with an RNA integrity number (RIN) higher than 7.0 were utilized after evaluating the quality and quantity of the extracted RNA using the NA Nano 6000 Assay Kit of the Bioanalyzer 2100 system (Agilent Technologies (St. Clara, CA, USA)). The index-coded samples were clustered using the TruSeq PE Cluster Kit v3-cBot-HS (Illumina (San Diego, CA, USA)) on a cBot Cluster Generation System, following the guidelines provided by the manufacturer. Raw reads were processed and quality controlled using FastQC v0.11.5. A reference genome index was created using HISAT2 v2.0.5. Then, the reads refined from the paired-end sequencing were aligned to the reference genome using the same tool. The DESeq2 package in the R environment was used to analyze differential gene expression, and genes showing an adjusted *p* value below 0.05 were classified as differently expressed genes (DEGs). To analyze the statistical enrichment of DEGs in GO–Biological Process (GO BP), Kyoto Encyclopedia of Genes and Genomes (KEGG), and Reactome, we utilized the clusterProfiler v3.8.1.

### 2.10. Western Blot Analysis

As described in our previous experimental approaches, we utilized RIPA lysis buffer enriched with protease and phosphatase inhibitors to extract total protein from liver tissue samples. The protein concentration was measured using a BCA protein assay kit. SDS–PAGE was used to separate protein amounts uniformly on 8% gels, which were then transferred to PVDF membranes. The specified proteins were incubated with primary antibodies at a temperature of 4 °C overnight. Following a washing process, the membranes were then incubated with secondary antibodies conjugated with HRP for 1 h at room temperature. The protein bands were observed utilizing an enhanced chemiluminescence (ECL) detection mechanism, and the intensity of the bands was measured using image analysis software. The expression levels of the target proteins were standardized by comparing them to the expression levels of the housekeeping proteins.

### 2.11. Statistical Analysis

GraphPad Prism 9.0 software was used for all the statistical computations. One-way ANOVA was used for statistical analysis in this study. The statistical results are expressed as the mean ± SD. Values of *p* < 0.05 were regarded statistically significant. 

## 3. Results

### 3.1. TIIA Improved Lipid Accumulation and Reduced Liver Injury

**Figure 1:**
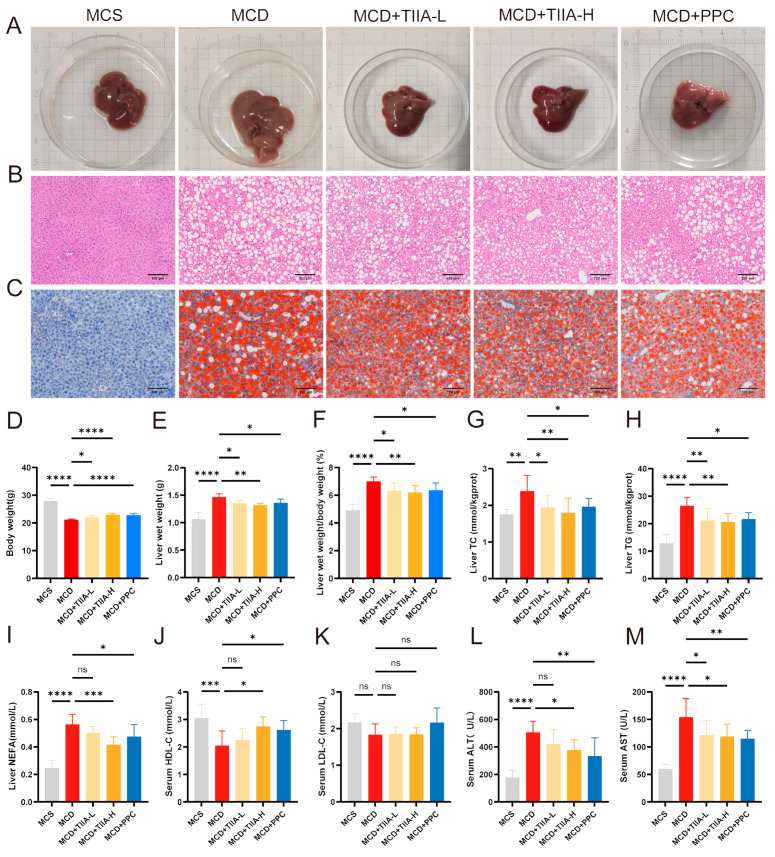
TIIA reduced hepatic lipid accumulation and normalized biochemical levels. (**A**–**C**) Representative liver images and histological images of liver tissue sections stained with HE and Oil Red O (100 µm; 200×). (**D**–**F**) Body weights and liver weights of the mice in each group. (**G**–**I**) TC, TG, and NEFA contents in the livers. (**J**–**M**) Serum concentrations of HDL-C, LDL-C, ALT, and AST. * *p* < 0.05; ** *p* < 0.01; *** *p* < 0.001; **** *p* < 0.0001. ns, not significant.

HE staining revealed extensive liver steatosis in the MCD group, and Oil Red O staining revealed a significant increase in liver lipid droplets in the MCD group. However, these conditions significantly improved after TIIA and PPC treatment (Figure 1A–C). As a typical feature of MCD feeding, mice in the MCD group dramatically lost weight, while TIIA and PPC treatment restored their weight to a certain extent (Figure 1D). The liver wet weight and liver wet weight/body weight; TC, TG, and NEFA levels; and serum ALT, and AST levels were significantly increased in the MCD group, accompanied by a decrease in HDL-C level, but there was no significant difference in LDL-C levels among the groups. However, TIIA and PPC treatment significantly reduced lipid levels in the liver and serum, indicating that TIIA and PPC restored normal hepatic lipid metabolism and attenuated hepatocellular injury (Figure 1E–M). Overall, efficacy in the MCD + TIIA-H group was generally better than that in the MCD + TIIA-L group.

### 3.2. TIIA Improved Liver Microcirculation and Fibrosis

**Figure 2:**
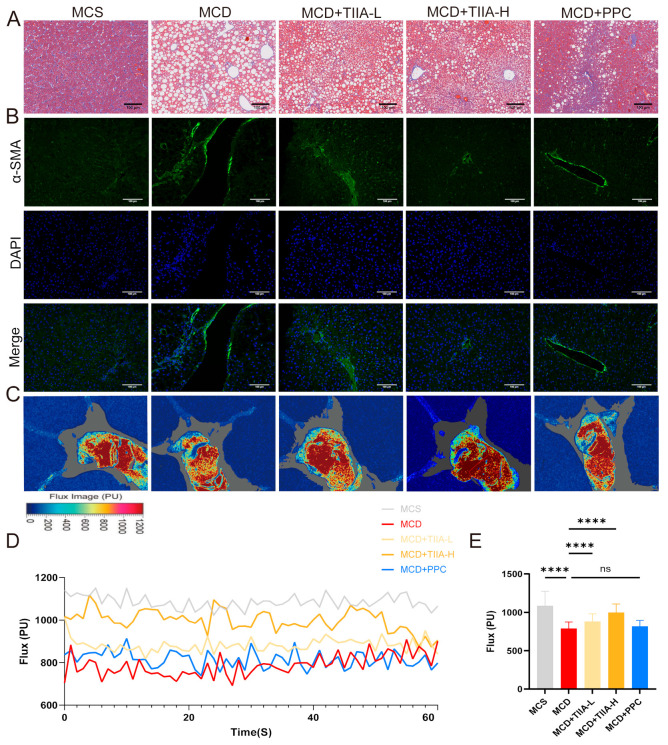
TIIA improved liver blood flow and reduced fibrosis. (**A**,**B**) Masson staining and α-SMA IF (100 µm; 200×). (**C**) Representative image of blood flux in superficial hepatic microcirculation. (**D**,**E**) Line and bar graphs of the mean hepatic superficial microcirculation blood flow in each group. **** *p* < 0.0001. ns, not significant.

Masson staining and α-smooth muscle actin (α-SMA) IF were used to assess liver fibrosis, and liver surface microblood flow scans and biochemical analysis were used to assess liver surface blood flow. Compared with those in the MCS group, collagen fibre in the MCD group exhibited higher expression of α-SMA, and blood flow was significantly lower. After treatment with TIIA and PPC, fibrosis was alleviated. TIIA restored liver blood flow, but PPC had no significant effect (Figure 2A–E).

### 3.3. TIIA Extensively Modulated the Hepatic Lipid Profile

**Figure 3:**
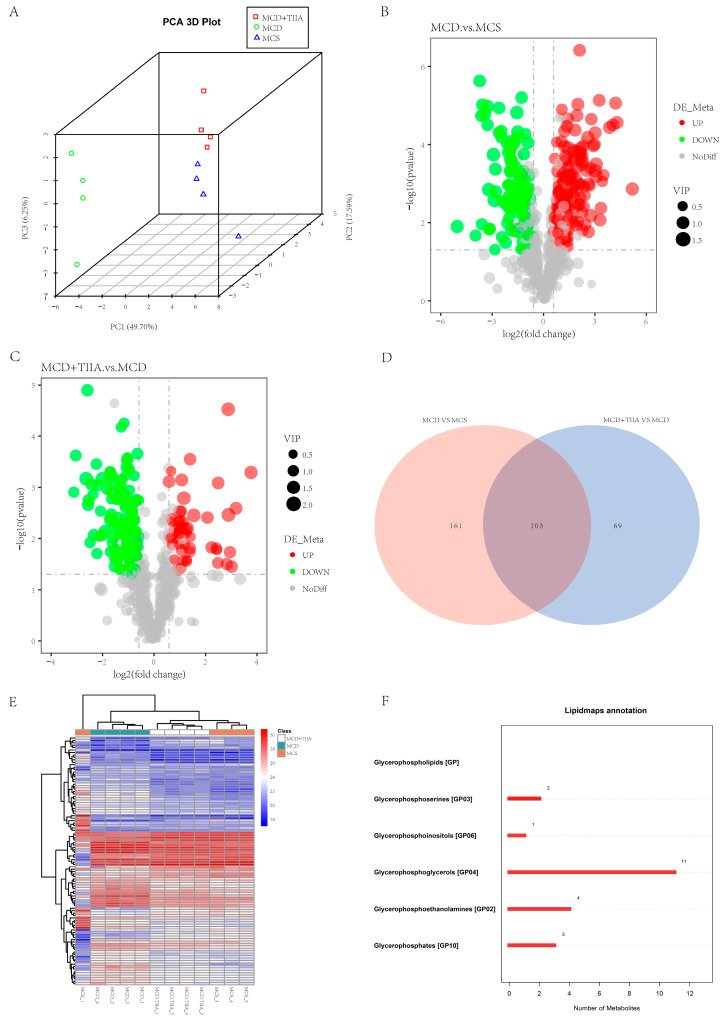
TIIA broadly modulates hepatic lipid metabolism. (**A**) Plots of the principal component analysis. (**B**–**E**) A volcano plot was created for the DALs, which were subsequently clustered to construct a Venn diagram and heatmap. (**F**) Lipidmaps annotation. VIP, Variable Importance in Projection.

To further verify the effect of TIIA on liver lipid metabolism, lipidomic analysis was used to evaluate the changes in the liver lipid profile in the MCS, MCD, and MCD + TIIA-H groups. Through PCA, we could see significant differences in lipids between the MCS, MCD, and MCD + TIIA groups, indicating that the NASH model was successful. Through the volcano map, we can see some lipid changes in the MCD group relative to the MCS group and the MCD + TIIA group. Using lipidomic analysis, we screened 103 lipid metabolites that were differentially altered between groups (Figure 3A–E). We functionally annotated these differential lipids and found that they were mainly enriched in the phospholipid metabolic pathway and mainly included glycerophosphoserines [GP03], glycerophosphoinositols [GP06], glycerophosphoglycerols [GP04], glycerophosphoethanolamines [GP02], and glycerophosphates [GP10] (Figure 3F).

### 3.4. TIIA Regulated ER Function through the PPAR Signalling Pathway

**Figure 4:**
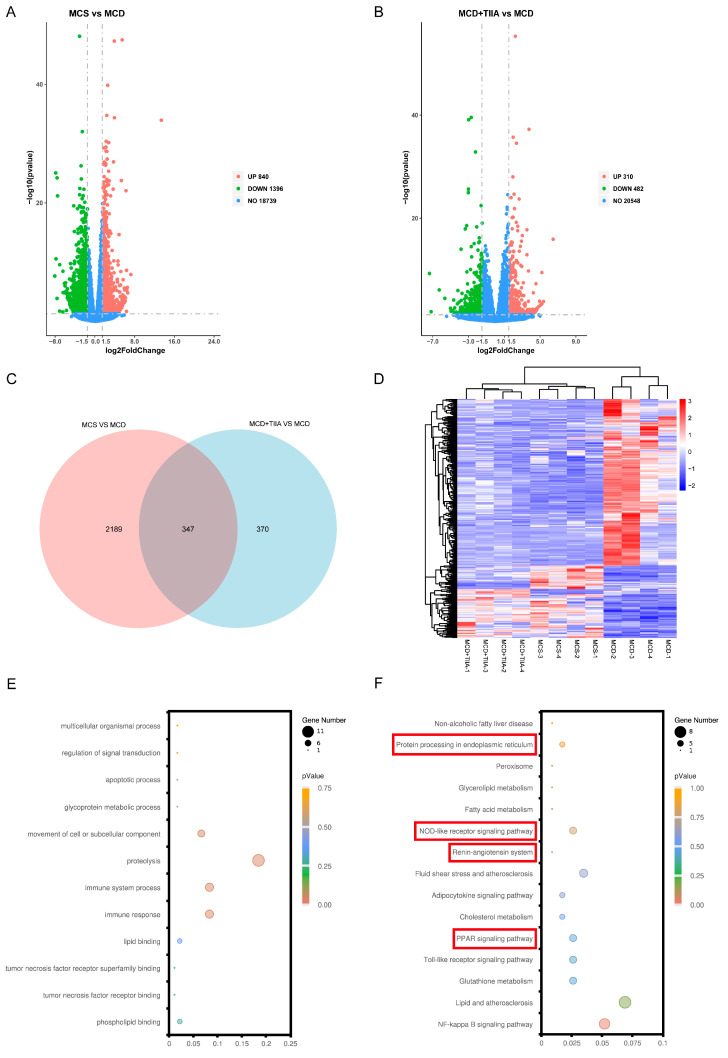
TIIA extensively regulates the liver transcriptome. (**A**–**D**) A volcano plot was created for the DEGs, which were clustered to create a Venn diagram and heatmap. (**E**–**F**) GO and KEGG analyses of the DEGs were performed, and a bubble chart was created.

RNA sequencing (RNA-seq) was used to reveal the underlying mechanism by which TIIA attenuates NASH. A total of 347 DEGs common to the MCS, MCD, and MCD + TIIA groups were selected for analysis (Figure 4A–D). The GO-biological process (GO:BP) analysis revealed that the DEGs were significantly enriched in the biological processes of immune responseand lipid metabolism regulation (Figure 4E). Similarly, the KEGG pathway analysis revealed several significantly enriched pathways, including the PPAR signalling pathway, protein processing in the ER, and the NOD-like receptor signalling pathway (Figure 4F). These results suggest that TIIA may exert its protective effect against NASH by participating in the regulation of these key biological processes and pathways.

### 3.5. TIIA Regulated ER Stress by Activating the PPARα/FGF21 Axis

**Figure 5:**
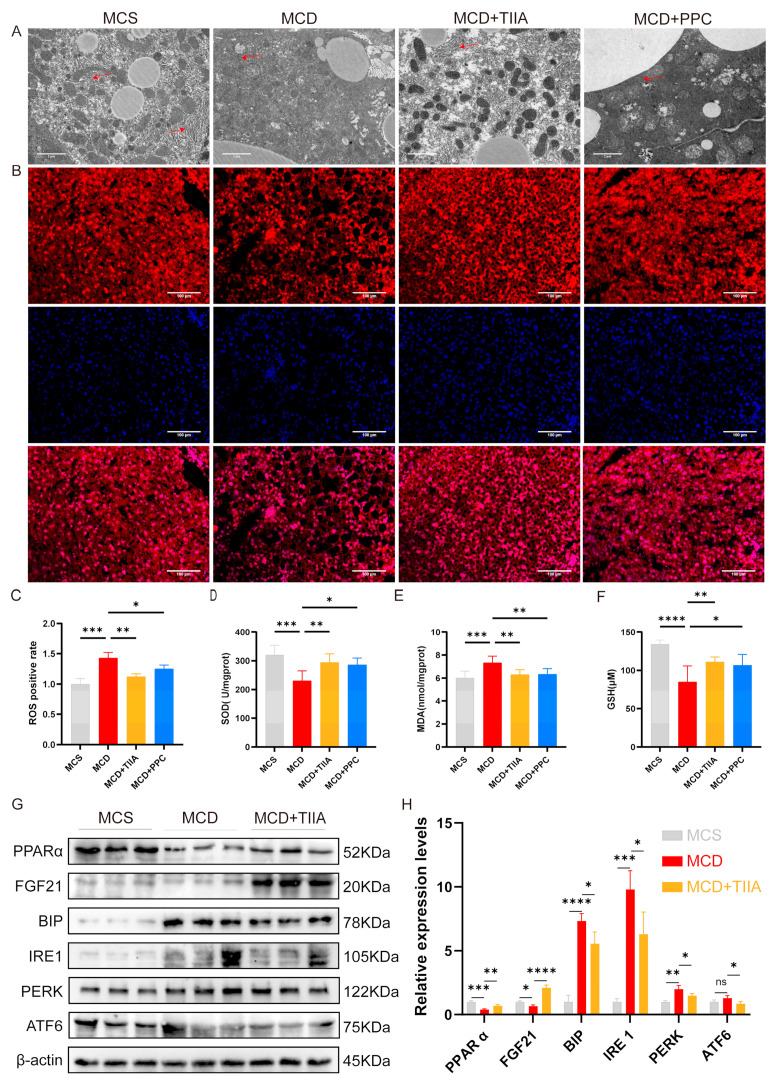
TIIA improves ER stress by regulating the PPARα/FGF21 axis. (**A**) Ultrathin liver sections observed via TEM (2 µm; 5000×). Red arrows indicate the endoplasmic reticulum. (**B**,**C**) ROS staining and relative expression levels (100 µm; 200×). **(D**–**F)** SOD, MDA, and GSH levels (n = 6). (**G**–**H**) The relative protein expression levels of PPARα, FGF21, BiP, IRE1, PERK, and ATF6 were assessed by means of Western blot analysis (n = 3). * *p* < 0.05; ** *p* < 0.01; *** *p* < 0.001; **** *p* < 0.0001. ns, not significant.

TEM revealed that the ER in the MCD group exhibited abnormal expansion and swelling, with the absence of a normal folding structure; however, these effects were normalized by TIIA and PPC treatment (Figure 5A). DHE staining and the measurement of SOD, MDA, and GSH levels in liver tissue were performed to evaluate the degree of oxidative stress. Compared with that in the MCS group, oxidative stress in the MCD group was significantly greater, and TIIA and PPC effectively reduced the oxidative stress level (Figure 5B–F). Compared with those in the MCS group, the expression levels of PPARα and FGF21 in the MCD group were significantly decreased, and the expression levels of BiP, IRE1, PERK, and ATF6 were significantly increased. TIIA treatment reversed these changes (Figure 5 G-H). These results suggest that TIIA may play a role in alleviating NASH by activating the PPARα/FGF21 axis to negatively regulate the UPR involved in ER stress. 

### 3.6. TIIA Alleviated Inflammation by Inhibiting NLRP3 Inflammasome Activation

**Figure 6:**
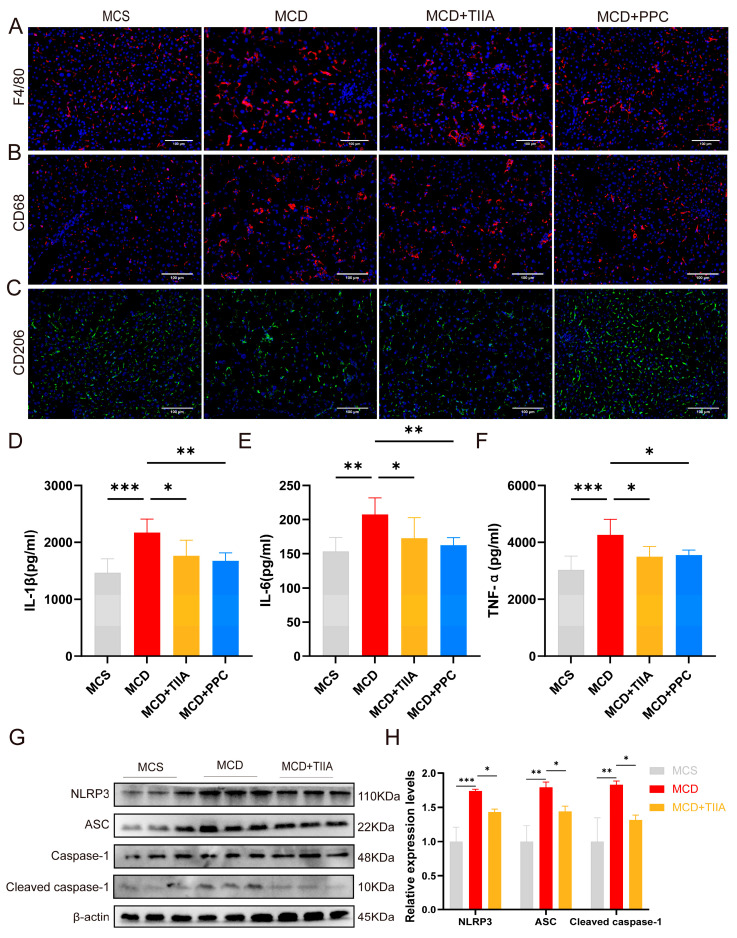
TIIA reduced inflammation and inhibited the activation of the NLRP3 inflammasome. (**A**) Micrographs of F4/80-stained hepatic sections (100 µm; 200×) (n = 3). (**B**,**C**) Micrographs of CD68 and CD206 staining within hepatic sections (100 µm; 200×) (n = 3). (**D**–**F**) Levels of liver IL-1β, IL-6, and TNF-α. (**G**–**H**) The relative protein expression levels of NLRP3, ASC, and Cleaved caspase-1 were assessed by means of Western blot analysis (n = 3). * *p* < 0.05; ** *p* < 0.01; *** *p* < 0.001. ns, not significant.

To evaluate the anti-inflammatory effect of TIIA, IF assays for F4/80, CD68, and CD206, and ELISAs for IL-1β, IL-6, and TNF-α were used to evaluate inflammatory levels in liver tissue. The MCD group showed increased inflammatory infiltration and expression of inflammatory factors in liver tissue, which were effectively reduced by TIIA and PPC treatment (Figure 6A–F). To elucidate the specific mechanism by which TIIA reduces the inflammatory response, the expression levels of the NOD-like receptor signalling pathway-related proteins NLRP3, ASC, and Caspase-1, which are enriched in the transcriptome, were assessed by means of Western blotting. TIIA reversed the increase in NLRP3, ASC, and Caspase-1 protein expression caused by the MCD diet (Figure 6G–H). These results suggest that TIIA may play an anti-inflammatory role by inhibiting the activation of the NLRP3 inflammasome.

## 4. Discussion

NASH is characterized by bullous steatosis with hepatocellular injury and inflammation [25]. The MCD diet is the classic approach for inducing NASH in animal models. This model has the advantages of a short modelling time, little influence from individual differences, and good homogeneity and stability of liver inflammation [26,27]. However, the MCD diet causes animals to lose weight without showing obesity and insulin resistance [28]. In this study, C57BL/6J mice were fed an MCD diet to establish a NASH model. HE and Oil Red O staining results showed that the MCD diet caused massive lipid accumulation in mouse hepatocytes, with round fat vacuoles and obvious balloon-like changes. TIIA reduced the fat accumulation induced by the MCD diet and significantly reduced the lipid levels in the liver and serum. The MCD diet lacks choline, which is involved in the synthesis of phospholipid membranes and is a vital essential nutrient for cells [29]. Therefore, PPC was selected as a positive control. Although there are currently no first-line drugs approved to treat NAFLD, PPC has been widely used by clinicians to treat patients suffered from NAFLD [30]. TIIA is more effective at reducing lipid accumulation than PPC. However, PPC is better at reducing liver damage.

Phospholipid metabolism plays an important role in regulating lipid, lipoprotein, and energy metabolism [31]. In patients, NASH is often accompanied by oxidative stress, and the oxidative phospholipids produced by oxidative stress have pro-inflammatory and pro-atherogenic effects. Targeting oxidative phospholipids may ameliorate NASH and its complications, including steatosis, inflammation, hepatocyte injury, cell death, fibrosis, and possible HCC formation [32]. Oxidative damage can enhance ER stress response, and UPR signal transduction is also regulated by the reversible modification of oxidative stress [33]. Lipidomic analysis suggested that TIIA significantly altered phospholipid metabolism in NASH mice. At the same time, TIIA can effectively reduce the oxidative stress state of NASH mice. TIIA may ameliorate NASH by reducing oxidized phospholipids produced by oxidative stress and inhibiting ER stress.

PPARα and FGF21 are mainly distributed in the liver and participate in the maintenance of hepatic lipid metabolism homeostasis [34,35]. In the liver, FGF21 can be directly activated by PPARα, forming the PPARα/FGF21 axis [36]. After activation, PPARα can directly bind to the FGF21 promoter region, thereby upregulating FGF21 expression [37]. Studies have shown that FGF21 expression is significantly reduced and liver fat accumulation is more obvious in PPARα knockout mice with NAFLD than in wild-type NAFLD mice [38]. The PPARα/FGF21 axis formed by PPARα and FGF21 is widely involved in maintaining liver lipid metabolism homeostasis, and regulating the PPARα/FGF21 axis may be an important way to prevent and treat NASH.

Studies have shown that the activation of the PPARα/FGF21 axis negatively regulates the UPR in the ER [39]. Unfolded proteins accumulate when ER stress occurs. As a marker protein of ER stress, BiP dissociates from IRE1, PERK, and ATF6 transmembrane transporters on the ER membrane, thereby affecting hepatic lipid metabolism. When the PPARα/FGF21 axis is activated, BiP can bind to and inactivate the transmembrane proteins IRE1, PERK, and ATF6 on the ER membrane, thereby inhibiting ER stress and exerting a negative regulatory effect [40]. Studies have confirmed that FGF21 knockout mice with NAFLD have more severe ER stress and steatosis in the liver than wild-type NAFLD mice [41]. ER stress and its related signalling pathways are closely related to the PPARα/FGF21 axis in the pathogenesis of NASH. By activating the PPARα/FGF21 axis, BiP, a marker protein of the ER, can be inactivated; thus, it negatively regulates the expression of IRE1, PERK, and ATF6 transmembrane transporters in the UPR, inhibits ER stress, and normalizes lipid metabolism. Transcriptome sequencing analysis and Western blot analysis also verified this hypothesis and confirmed that TIIA may negatively regulate the UPR in response to ER stress by activating the PPARα/FGF21 axis to ameliorate NASH.

**Figure 7:**
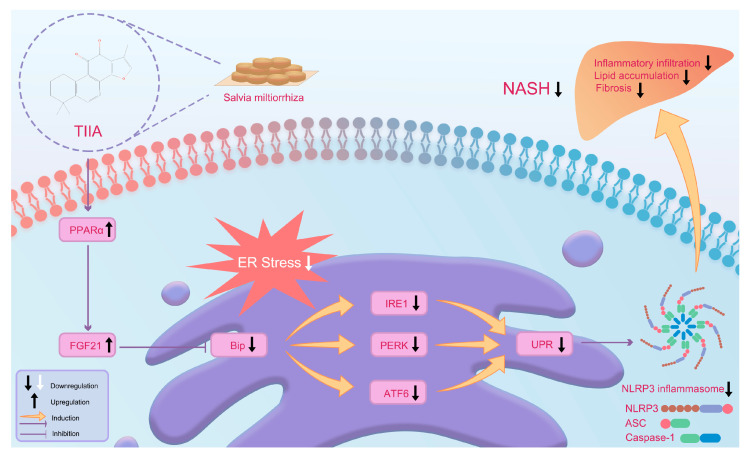
Potential mechanisms of TIIA in NASH mice. TIIA ameliorates NASH by inhibiting ER stress-induced UPR and NLRP3 inflammasome activation by activating the PPARα/FGF21 axis.

ER stress can induce the activation of the NLRP3 inflammasome [42]. Chronic inflammation is a core component of the pathogenesis of NASH, and it is also a key factor in the progression of simple obesity to NASH, cirrhosis, and even liver cancer [43]. NLRP3 inflammasome activation was confirmed to be indispensable for the development of NASH [44]. The NLRP3 inflammasome is a protein complex composed of NLRP3, apoptosis-associated speck-like protein (ASC), and a caspase-1 precursor that is widely expressed in a variety of cells [45]. The NLRP3 inflammasome can promote the expression and release of specific inflammatory factors, leading to liver inflammation and damage and subsequently affecting the occurrence and progression of NASH [46]. IF and ELISAs confirmed that TIIA could reduce the level of inflammation in the liver tissue of NASH mice. Western blot analysis further confirmed that TIIA might play an anti-inflammatory role by inhibiting the production of the NLRP3 inflammasome. After the activation of the NLRP3 inflammasome, hepatocytes will undergo pyroptosis and release NLRP3 inflammasome protein. Hepatic stellate cells will phagocytose extracellular NLRP3 inflammasome particles, resulting in increased secretion of inflammatory cytokines and α-SMA expression, further amplifying and perpetuating inflammasome-driven fibrosis [47]. Our study further confirmed that TIIA can effectively improve liver fibrosis. The potential mechanism of TIIA in NASH mice is illustrated in Figure 7.

Lipid deposition and balloon-like degeneration in hepatocytes squeezes the hepatic sinusoid, reduces blood flow in the hepatic sinusoid, and produces corresponding physical shear stress, which affects the homeostasis of microcirculation in the hepatic sinusoid, seriously affects the metabolism of hepatocytes, and causes abnormal liver function [48,49]. We tested the liver surface blood flow of each group of mice, and found that TIIA could effectively improve liver microcirculation, but PPC had no obvious effect. It was demonstrated that TIIA did not improve liver microcirculation by improving lipid accumulation alone. Studies have shown that a local renin–angiotensin system exists in the liver and have revealed its major components [50]. Renin inhibitors have also been shown to reduce hepatic steatosis and fibrosis in NAFLD mice [51]. The renin–angiotensin system pathway was also enriched according to KEGG transcriptome analysis. Masson staining and α-SMA IF showed that TIIA could effectively slow down the progression of NASH to liver fibrosis. Salvia miltiorrhiza is a commonly used natural medicine for treating cardiovascular diseases. TIIA is an active component extracted from Salvia miltiorrhiza. TIIA has been shown to have anti-inflammatory, anticoagulant, antithrombotic, and pro-angiogenic effects [52]. Whether TIIA can improve the liver fibrosis of NASH mice by improving microcirculation, increasing blood flow, and the regulating renin–angiotensin system while improving lipid accumulation is worth further study.

PPARs include PPARα, PPARβ/δ, and PPARγ. Transcriptomic results suggested that TIIA plays a role through the PPAR pathway, and our study confirmed that TIIA may play an anti-NASH role by regulating PPARα, but we did not confirm whether it also plays a role through PPARβ/δ and PPARγ. At present, most of the PPAR agonists used in the treatment of NAFLD are panagonists [53,54,55,56]. TIIA has also been shown to improve lipid accumulation and oxidative stress in NAFLD rats by targeting PPARγ and TLR4 [57]. Therefore, whether TIIA plays an anti-NASH role as a pan-PPAR agonist needs further study.

## 5. Conclusions

In summary, TIIA can reduce liver lipid accumulation, inflammatory infiltration, and fibrosis in NASH mice, and this effect may be achieved by activating the PPARα/FGF21 axis to negatively regulate the ER stress-induced UPR and inhibit NLRP3 inflammasome activation.

## Data Availability

The raw data supporting the conclusions of this article will be made available by the authors on request.

## References

[1] Harrison S.A., Allen A.M., Dubourg J., Noureddin M., Alkhouri N. (2023). Challenges and opportunities in NASH drug development. Nat. Med..

[2] Kanwal F., Shubrook J.H., Younossi Z., Natarajan Y., Bugianesi E., Rinella M.E., Harrison S.A., Mantzoros C., Pfotenhauer K., Klein S. (2021). Preparing for the NASH Epidemic: A Call to Action. Gastroenterology.

[3] Loomba R., Friedman S.L., Shulman G.I. (2021). Mechanisms and disease consequences of nonalcoholic fatty liver disease. Cell.

[4] Musso G., Saba F., Cassader M., Gambino R. (2023). Lipidomics in pathogenesis, progression and treatment of nonalcoholic steatohepatitis (NASH): Recent advances. Prog. Lipid Res..

[5] Ye L., Zhao D., Xu Y., Lin J., Xu J., Wang K., Ye Z., Luo Y., Liu S., Yang H. (2021). LncRNA-Gm9795 promotes inflammation in non-alcoholic steatohepatitis via NF-κB/JNK pathway by endoplasmic reticulum stress. J. Transl. Med..

[6] Xu X., Poulsen K.L., Wu L., Liu S., Miyata T., Song Q., Wei Q., Zhao C., Lin C., Yang J. (2022). Targeted therapeutics and novel signaling pathways in non-alcohol-associated fatty liver/steatohepatitis (NAFL/NASH). Signal Transduct. Target. Ther..

[7] Paternostro R., Trauner M. (2022). Current treatment of non-alcoholic fatty liver disease. J. Intern. Med..

[8] Guo C., Li Q., Chen R., Fan W., Zhang X., Zhang Y., Guo L., Wang X., Qu X., Dong H. (2023). Baicalein alleviates non-alcoholic fatty liver disease in mice by ameliorating intestinal barrier dysfunction. Food Funct..

[9] Lan T., Yu Y., Zhang J., Li H., Weng Q., Jiang S., Tian S., Xu T., Hu S., Yang G. (2021). Cordycepin Ameliorates Nonalcoholic Steatohepatitis by Activation of the AMP-Activated Protein Kinase Signaling Pathway. Hepatology.

[10] Li H., Hu P., Zou Y., Yuan L., Xu Y., Zhang X., Luo X., Zhang Z. (2023). Tanshinone IIA and hepatocellular carcinoma: A potential therapeutic drug. Front. Oncol..

[11] Wang J., Zhang Y., Feng X., Du M., Li S., Chang X., Liu P. (2023). Tanshinone IIA alleviates atherosclerosis in LDLR(-/-) mice by regulating efferocytosis of macrophages. Front. Pharmacol..

[12] Shi M.J., Dong B.S., Yang W.N., Su S.B., Zhang H. (2019). Preventive and therapeutic role of Tanshinone IIA in hepatology. Biomed. Pharmacother..

[13] Li M., Li H., Liu H., Lai X., Xing W. (2022). Efficacy and safety of eight types Salvia miltiorrhiza injections in the treatment of unstable angina pectoris: A network meta-analysis. Front. Pharmacol..

[14] Jung I., Kim H., Moon S., Lee H., Kim B. (2020). Overview of Salvia miltiorrhiza as a Potential Therapeutic Agent for Various Diseases: An Update on Efficacy and Mechanisms of Action. Antioxidants.

[15] Xu L., Zhang Y., Ji N., Du Y., Jia T., Wei S., Wang W., Zhang S., Chen W. (2022). Tanshinone IIA regulates the TGF-beta1/Smad signaling pathway to ameliorate non-alcoholic steatohepatitis-related fibrosis. Exp. Ther. Med..

[16] Xu L., Liu X., Jia T., Sun Y., Du Y., Wei S., Wang W., Zhang Y., Chen W., Zhang S. (2022). Tanshinone IIA Ameliorates Nonalcoholic Steatohepatitis in Mice by Modulating Neutrophil Extracellular Traps and Hepatocyte Apoptosis. Evid. Based Complement. Alternat. Med..

[17] Wang J., Hu R., Yin C., Xiao Y. (2020). Tanshinone IIA reduces palmitate-induced apoptosis via inhibition of endoplasmic reticulum stress in HepG2 liver cells. Fundam. Clin. Pharmacol..

[18] Farese R.J., Walther T.C. (2023). Glycerolipid Synthesis and Lipid Droplet Formation in the Endoplasmic Reticulum. Cold Spring Harb. Perspect. Biol..

[19] Sniegocka M., Liccardo F., Fazi F., Masciarelli S. (2022). Understanding ER homeostasis and the UPR to enhance treatment efficacy of acute myeloid leukemia. Drug Resist. Updat..

[20] Anastasia I., Ilacqua N., Raimondi A., Lemieux P., Ghandehari-Alavijeh R., Faure G., Mekhedov S.L., Williams K.J., Caicci F., Valle G. (2021). Mitochondria-rough-ER contacts in the liver regulate systemic lipid homeostasis. Cell Rep..

[21] Ajoolabady A., Kaplowitz N., Lebeaupin C., Kroemer G., Kaufman R.J., Malhi H., Ren J. (2023). Endoplasmic reticulum stress in liver diseases. Hepatology.

[22] Celik C., Lee S., Yap W.S., Thibault G. (2023). Endoplasmic reticulum stress and lipids in health and diseases. Prog. Lipid. Res..

[23] Wiseman R.L., Mesgarzadeh J.S., Hendershot L.M. (2022). Reshaping endoplasmic reticulum quality control through the unfolded protein response. Mol. Cell.

[24] Lebeaupin C., Vallee D., Hazari Y., Hetz C., Chevet E., Bailly-Maitre B. (2018). Endoplasmic reticulum stress signalling and the pathogenesis of non-alcoholic fatty liver disease. J. Hepatol..

[25] Younossi Z., Anstee Q.M., Marietti M., Hardy T., Henry L., Eslam M., George J., Bugianesi E. (2018). Global burden of NAFLD and NASH: Trends, predictions, risk factors and prevention. Nat. Rev. Gastroenterol. Hepatol..

[26] Alshawsh M.A., Alsalahi A., Alshehade S.A., Saghir S., Ahmeda A.F., Al Z.R., Mahmoud A.M. (2022). A Comparison of the Gene Expression Profiles of Non-Alcoholic Fatty Liver Disease between Animal Models of a High-Fat Diet and Methionine-Choline-Deficient Diet. Molecules.

[27] Zhang X., Fan L., Wu J., Xu H., Leung W.Y., Fu K., Wu J., Liu K., Man K., Yang X. (2019). Macrophage p38alpha promotes nutritional steatohepatitis through M1 polarization. J. Hepatol..

[28] Reid D.T., Eksteen B. (2015). Murine models provide insight to the development of non-alcoholic fatty liver disease. Nutr. Res. Rev..

[29] Gallo M., Gamiz F. (2023). Choline: An Essential Nutrient for Human Health. Nutrients.

[30] Zhang J., Zang X., Lv J., Zhang Y., Lv Z., Yu M. (2023). Changes in Lipidomics, Metabolomics, and the Gut Microbiota in CDAA-Induced NAFLD Mice after Polyene Phosphatidylcholine Treatment. Int. J. Mol. Sci..

[31] van der Veen J.N., Kennelly J.P., Wan S., Vance J.E., Vance D.E., Jacobs R.L. (2017). The critical role of phosphatidylcholine and phosphatidylethanolamine metabolism in health and disease. Biochim. Biophys. Acta Biomembr..

[32] Sun X., Seidman J.S., Zhao P., Troutman T.D., Spann N.J., Que X., Zhou F., Liao Z., Pasillas M., Yang X. (2020). Neutralization of Oxidized Phospholipids Ameliorates Non-alcoholic Steatohepatitis. Cell Metab..

[33] Xiong S., Chng W.J., Zhou J. (2021). Crosstalk between endoplasmic reticulum stress and oxidative stress: A dynamic duo in multiple myeloma. Cell. Mol. Life Sci..

[34] Hu P., Li K., Peng X., Kan Y., Li H., Zhu Y., Wang Z., Li Z., Liu H.Y., Cai D. (2023). Nuclear Receptor PPARalpha as a Therapeutic Target in Diseases Associated with Lipid Metabolism Disorders. Nutrients.

[35] Lu W., Li X., Luo Y. (2021). FGF21 in obesity and cancer: New insights. Cancer Lett..

[36] Piccinin E., Moschetta A. (2016). Hepatic-specific PPARalpha-FGF21 action in NAFLD. Gut.

[37] Tezze C., Romanello V., Sandri M. (2019). FGF21 as Modulator of Metabolism in Health and Disease. Front. Physiol..

[38] Montagner A., Polizzi A., Fouche E., Ducheix S., Lippi Y., Lasserre F., Barquissau V., Regnier M., Lukowicz C., Benhamed F. (2016). Liver PPARalpha is crucial for whole-body fatty acid homeostasis and is protective against NAFLD. Gut.

[39] Inagaki T. (2015). Research Perspectives on the Regulation and Physiological Functions of FGF21 and its Association with NAFLD. Front. Endocrinol..

[40] Kim S.H., Kim K.H., Kim H.K., Kim M.J., Back S.H., Konishi M., Itoh N., Lee M.S. (2015). Fibroblast growth factor 21 participates in adaptation to endoplasmic reticulum stress and attenuates obesity-induced hepatic metabolic stress. Diabetologia.

[41] Tanaka N., Takahashi S., Zhang Y., Krausz K.W., Smith P.B., Patterson A.D., Gonzalez F.J. (2015). Role of fibroblast growth factor 21 in the early stage of NASH induced by methionine- and choline-deficient diet. Biochim. Biophys. Acta.

[42] Lu X., Huang H., Fu X., Chen C., Liu H., Wang H., Wu D. (2022). The Role of Endoplasmic Reticulum Stress and NLRP3 Inflammasome in Liver Disorders. Int. J. Mol. Sci..

[43] Wang X., He Q., Zhou C., Xu Y., Liu D., Fujiwara N., Kubota N., Click A., Henderson P., Vancil J. (2023). Prolonged hypernutrition impairs TREM2-dependent efferocytosis to license chronic liver inflammation and NASH development. Immunity.

[44] Deng Y.F., Xu Q.Q., Chen T.Q., Ming J.X., Wang Y.F., Mao L.N., Zhou J.J., Sun W.G., Zhou Q., Ren H. (2022). Kinsenoside alleviates inflammation and fibrosis in experimental NASH mice by suppressing the NF-kappaB/NLRP3 signaling pathway. Phytomedicine.

[45] Pavillard L.E., Marin-Aguilar F., Bullon P., Cordero M.D. (2018). Cardiovascular diseases, NLRP3 inflammasome, and western dietary patterns. Pharmacol. Res..

[46] Ramos-Tovar E., Muriel P. (2023). NLRP3 inflammasome in hepatic diseases: A pharmacological target. Biochem. Pharmacol..

[47] Gaul S., Leszczynska A., Alegre F., Kaufmann B., Johnson C.D., Adams L.A., Wree A., Damm G., Seehofer D., Calvente C.J. (2021). Hepatocyte pyroptosis and release of inflammasome particles induce stellate cell activation and liver fibrosis. J. Hepatol..

[48] Li J., Zhang X., Tian J., Li J., Li X., Wu S., Liu Y., Han J., Ye F. (2023). CX08005, a Protein Tyrosine Phosphatase 1B Inhibitor, Attenuated Hepatic Lipid Accumulation and Microcirculation Dysfunction Associated with Nonalcoholic Fatty Liver Disease. Pharmaceuticals.

[49] Sun X., Harris E.N. (2020). New aspects of hepatic endothelial cells in physiology and nonalcoholic fatty liver disease. Am. J. Physiol. Cell Physiol..

[50] Hartl L., Rumpf B., Domenig O., Simbrunner B., Paternostro R., Jachs M., Poglitsch M., Marculescu R., Trauner M., Reindl-Schwaighofer R. (2023). The systemic and hepatic alternative renin-angiotensin system is activated in liver cirrhosis, linked to endothelial dysfunction and inflammation. Sci. Rep..

[51] Lee K.C., Wu P.S., Lin H.C. (2023). Pathogenesis and treatment of non-alcoholic steatohepatitis and its fibrosis. Clin. Mol. Hepatol..

[52] Guo R., Li L., Su J., Li S., Duncan S.E., Liu Z., Fan G. (2020). Pharmacological Activity and Mechanism of Tanshinone IIA in Related Diseases. Drug Des. Devel. Ther..

[53] Francque S.M., Bedossa P., Ratziu V., Anstee Q.M., Bugianesi E., Sanyal A.J., Loomba R., Harrison S.A., Balabanska R., Mateva L. (2021). A Randomized, Controlled Trial of the Pan-PPAR Agonist Lanifibranor in NASH. N. Engl. J. Med..

[54] Gawrieh S., Noureddin M., Loo N., Mohseni R., Awasty V., Cusi K., Kowdley K.V., Lai M., Schiff E., Parmar D. (2021). Saroglitazar, a PPAR-alpha/gamma Agonist, for Treatment of NAFLD: A Randomized Controlled Double-Blind Phase 2 Trial. Hepatology.

[55] Siddiqui M.S., Idowu M.O., Parmar D., Borg B.B., Denham D., Loo N.M., Lazas D., Younes Z., Sanyal A.J. (2021). A Phase 2 Double Blinded, Randomized Controlled Trial of Saroglitazar in Patients With Nonalcoholic Steatohepatitis. Clin. Gastroenterol. Hepatol..

[56] Nakajima A., Eguchi Y., Yoneda M., Imajo K., Tamaki N., Suganami H., Nojima T., Tanigawa R., Iizuka M., Iida Y. (2021). Randomised clinical trial: Pemafibrate, a novel selective peroxisome proliferator-activated receptor alpha modulator (SPPARMalpha), versus placebo in patients with non-alcoholic fatty liver disease. Aliment. Pharmacol. Ther..

[57] Huang L., Ding W., Wang M.Q., Wang Z.G., Chen H.H., Chen W., Yang Q., Lu T.N., Yang Q., He J.M. (2019). Tanshinone IIA ameliorates non-alcoholic fatty liver disease through targeting peroxisome proliferator-activated receptor gamma and toll-like receptor 4. J. Int. Med. Res..

